# Establishment of liver tumor cell lines from atherogenic and high fat diet fed hepatitis C virus transgenic mice

**DOI:** 10.1038/s41598-021-92128-9

**Published:** 2021-06-22

**Authors:** Takayoshi Shirasaki, Kazuhisa Murai, Masao Honda, Hikari Okada, Yuika Innami, Atsumu Yamada, Tetsuro Shimakami, Kazunori Kawaguchi, Taro Yamashita, Yoshio Sakai, Shuichi Kaneko

**Affiliations:** 1 Department of Gastroenterology, Graduate School of Medicine, Kanazawa University, Takara-machi 13-1, Kanazawa, 920-8641 Japan; 2 Department of Laboratory Medicine, Graduate School of Health Medicine, Kanazawa University, Kanazawa, Japan

**Keywords:** Cancer, Cell biology, Microbiology

## Abstract

A syngeneic mouse model bearing a transplanted tumor is indispensable for the evaluation of the efficacy of immune checkpoint inhibitors (ICIs). However, few syngeneic mouse models of liver cancer are available. We established liver tumor cell lines (MHCF1 and MHCF5) from hepatitis C virus transgenic mice fed an atherogenic high-fat diet. MHCF1 and MHCF5 were successfully transplanted into the subcutaneous space of syngeneic C57BL/6 mice, in addition, they efficiently developed orthotopic tumors in the liver of syngeneic C57BL/6 mice. MHCF5 grew rapidly and showed a more malignant phenotype compared with MHCF1. Histologically, MHCF1-derived tumors were a combined type of hepatocellular carcinoma and MHCF5-derived tumors showed a sarcomatous morphology. Interestingly, MHCF1 and MHCF5 showed different sensitivity against an anti-PD1 antibody and MHCF5-derived tumors were resistant to this antibody. CD8 T cells infiltrated the MHCF1-derived tumors, but no CD8 T cells were found within the MHCF5-derived tumors. Gene expression profiling and whole-exon sequencing revealed that MHCF5 displayed the features of an activated cancer stem cell-like signature of sonic hedgehog and *Wnt* signaling. Therefore, these cell lines could be useful for the identification of new biomarkers and molecular mechanisms of ICI resistance and the development of new drugs against liver cancer.

## Introduction

Liver cancer is one of the leading causes of death from cancer and is frequently accompanied with intrahepatic recurrence, vascular invasion, and distant metastasis. The development of treatment against advanced liver cancer has encountered various hurdles, but recently developed advanced tyrosine kinase inhibitors (TKIs) such as sorafenib, regorafenib, lenvatinib, and cabozantinib are expected to improve the prognosis of patients with advanced hepatocellular carcinoma (HCC)^1,2^.

In parallel, the discovery of immune checkpoint molecules, such as programmed cell death 1 and cytotoxic T-lymphocyte antigen-4, has enabled a new strategy of cancer immunotherapy, and immune checkpoint inhibitors (ICIs) have dramatically improved the prognosis of various cancers. However, single-agent ICI trials for HCC or cholangiocellular carcinoma (CCC) have been relatively disappointing^3^. The recently developed combination therapy of atezolizumab (anti-PD-L1 antibody) and bevacizumab (anti-VEGF antibody) resulted in better overall survival for unresectable HCC^4^. Therefore, it is now important to develop an effective combination therapy with ICIs and chemotherapy including biologic drugs or TKIs^3,5^.

To investigate the molecular mechanism of drug resistance in vivo, a mouse model of liver cancer is indispensable^6^. Although xenograft models of human cancer cell lines in immune-deficient NOD-SCID mice are commonly used for the rapid evaluation of tumor growth, such a model is not suitable for the evaluation of ICIs because the tumor recipient mouse lacks acquired anti-tumor immunity. Thus, a syngeneic tumor graft model is required in which genetically matched mouse tumor cells can grow. However, at present, there are few syngeneic tumor graft models of liver cancer.

In this study, we established new liver tumor cell lines from hepatitis C virus (HCV) transgenic (Tg) mice fed an atherogenic and high-fat diet (Ath + HFD). The obtained cell lines were transplanted into syngeneic immune-competent C57BL/6 mice and formed subcutaneous tumor or orthotopic tumors in the liver. Our model might be useful for the development of new combinations of ICIs and anti-cancer drugs against liver cancer.

## Results

### Establishment of liver tumor cell lines from different mouse HCC models

**Figure 1:**
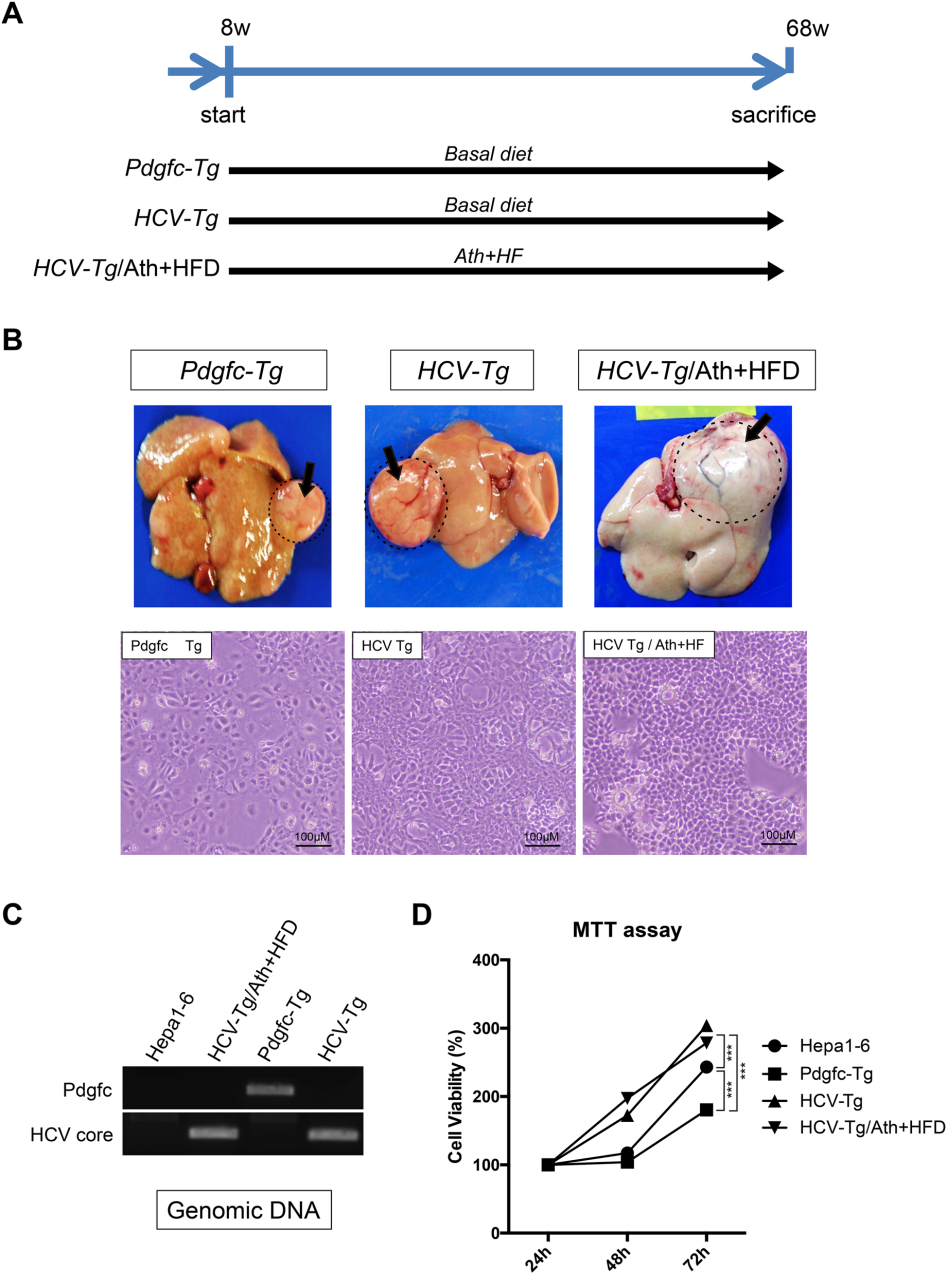
Establishment of liver tumor cell lines derived from Pdgfc-Tg, HCV-Tg, and HCV-Tg/Ath + HFD mice*.* (**A**) Feeding schedule of each group of mice. (**B**) Developed liver tumors in Pdgfc-Tg, HCV-Tg, and HCV-Tg/Ath + HFD mice (upper), and established cell lines from each tumor (lower). (**C**) PCR amplification of the integrated genes (*Pdgfc* and HCV). Full-length agarose gels are presented in Supplemental Fig. 1. (**D**) MTT assay of each cell line derived from Pdgfc-Tg, HCV-Tg, and HCV-Tg/Ath + HFD mice and the Hepa1-6 cell line.

We utilized two genetically engineered mouse HCC models, platelet-derived growth factor c (Pdgfc)-Tg^7^ and HCV-Tg mice^8^. Pdgfc-Tg mice express *Pdgfc* under the control of the albumin promoter and develop hepatic fibrosis and HCC^7^. HCV-Tg mice express the full-length coding region of genotype 1b HCV polyprotein and develop steatosis and HCC^8^. Tg mice were fed a basal diet and sacrificed at 68 weeks. A group of HCV-Tg mice was fed Ath + HFD for 60 weeks (HCV-Tg/Ath + HFD) (Fig. 1A). At 68 weeks, 7 of 9 (77.8%) Pdgfc-Tg mice developed liver tumors, 2 of 11 (18.2%) HCV-Tg mice developed liver tumors, and 9 of 19 (47.4%) HCV-Tg/Ath + HFD mice developed liver tumors (data not shown). The tumor cells were dissociated and seeded on collagen-coated dishes and grown and passaged several times. We established stable cell lines derived from Pdgfc-Tg, HCV-Tg, and HCV-Tg/Ath + HFD tumors (Fig. 1B). To confirm the origin of these cells, the integrated trans-genes were amplified by PCR using genomic DNA. The expected size of each fragment of *Pdgfc* and HCV was detected (Fig. 1C and Supplemental Fig. 1). The growth of these cell lines was compared with Hepa1-6 cells, a C57L-derived mouse liver HCC cell line. An MTT assay showed that HCV-Tg- and HCV-Tg/Ath + HFD-derived cells grew faster than Hepa1-6 cells, and Pdgfc-Tg-derived cells grew slower than Hepa1-6 cells (Fig. 1D).

### Allograft and syngeneic tumor models of established mouse liver tumor cell lines

**Figure 2:**
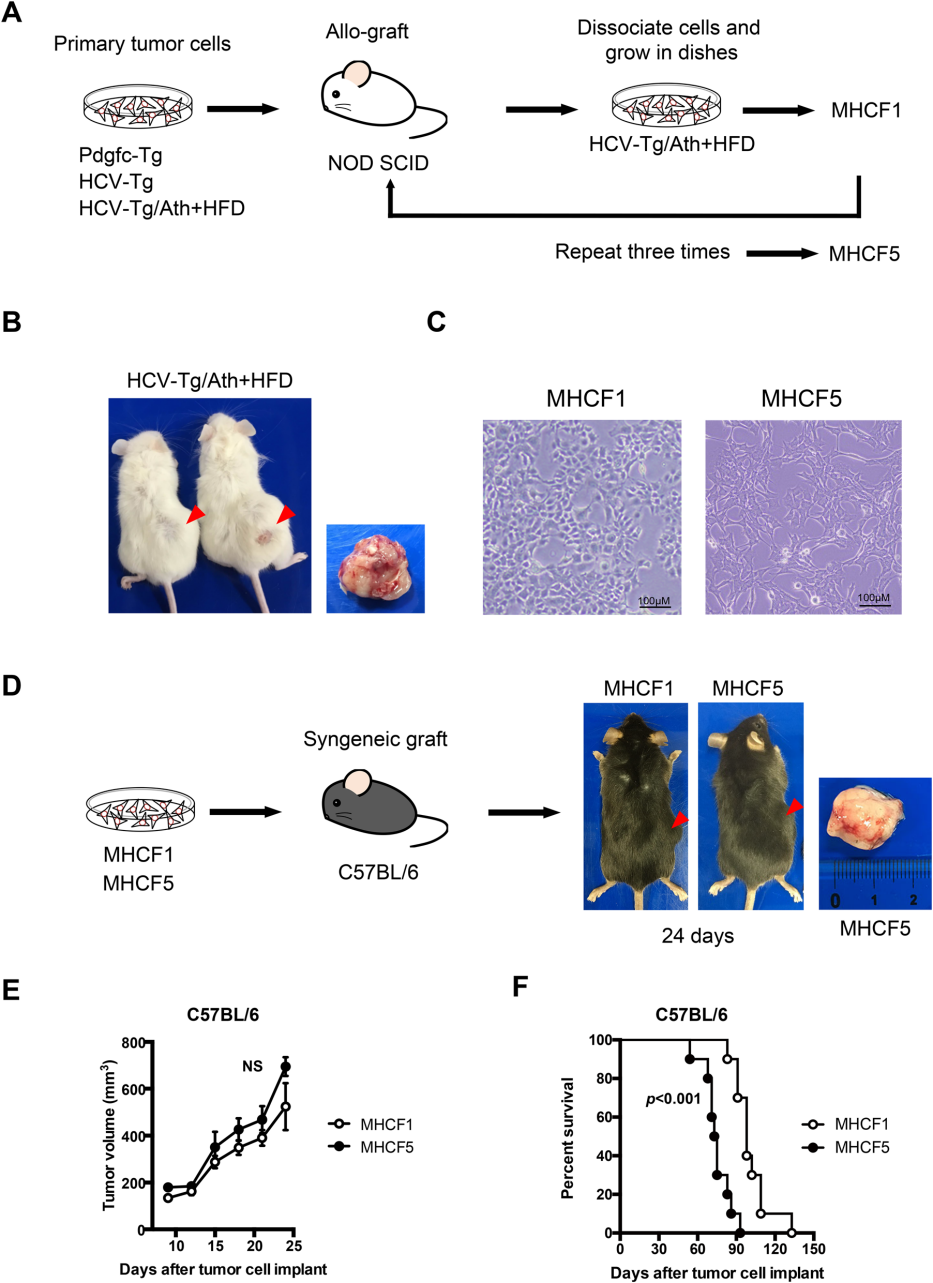
Establishment of MHCF1 and MHCF5 cells for syngeneic liver tumor mouse models. (**A**) Established liver tumor cell lines derived from Pdgfc-Tg, HCV-Tg, and HCV-Tg/Ath + HFD mice were transplanted subcutaneously into immune-deficient NOD-SCID mice. Cells were isolated from the developed tumors and maintained on culture dishes (MHCF1 cells). MHCF1 cells were repeatedly transplanted into NOD-SCID mice, and the cells were isolated again from the developed tumors. After three cycles of this procedure, MHCF5 cells were obtained. (**B**) The cell line derived from HCV-Tg/Ath + HFD mice developed into tumors in NOD-SCID mice. (**C**) Morphology of MHCF1 and MHCF5 cells on culture dishes. (**D**) MHCF1 and MHCF5 cells were successfully transplanted into immune-competent syngeneic C57BL/6 mice. (**E**) MTT assay of MHCF1 and MHCF5 cells. (**F**) Percentage survival of MHCF1- and MHCF5-transplanted mice.

The established cell lines were transplanted subcutaneously into immune-deficient NOD-SCID mice. Among the three cell lines (derived from HCV-Tg, HCV-Tg/Ath + HFD, and Pdgfc-Tg tumors), one cell line, an HCV-Tg/Ath + HFD-derived cell line, developed into tumors in NOD-SCID mice (Fig. 2A,B). The tumor cells were dissociated and grown on dishes. The cells were grown and passaged several times. We confirmed the cells were positive for albumin (*Alb*) and alpha-feto protein (*Afp*) mRNA by RT-PCR after several passages. We named this cell line mouse liver tumor cells derived from HCV full-length Tg No. 1 (MHCF1) (Fig. 2C). MHCF1 cells were then transplanted subcutaneously into syngeneic C57BL/6 mice. MHCF1 cells developed into tumors in C57BL/6 mice (Fig. 2D), whereas Hepa1-6 cells did not (data not shown).

To obtain cell lines that could adapt to the microenvironment of the in vivo mouse model, MHCF1 cells were repeatedly transplanted into NOD-SCID mice, and the developed tumors were dissociated and passaged on culture dishes several times. We performed these procedures three times (Fig. 2A). After three cycles, the obtained cells were seeded on 96-well plates with limiting dilution and 12 clones were obtained. All clones were negative for *Alb* and *Afp* mRNA. Six of the 12 clones were transplanted subcutaneously into syngeneic C57BL/6 mice and all clones developed into tumors. We selected one fast growing clone and named it MHCF5 (Fig. 2D). A trace amount of HCV-RNA was detected in MHCF1 and MHCF5 cells by RT-PCR; however, HCV protein was not detected in either cell line by western blotting (Supplemental Fig. 2). Although the volume of MHCF1- and MHCF5-derived tumors in C57BL/6 mice did not differ significantly within 25 days (Fig. 2E), the overall survival of MHCF5 tumor-bearing mice was significantly shorter than that of MHCF1 tumor-bearing mice (Fig. 2F).

### Histopathological features of MHCF1- and MHCF5-derived tumors

**Figure 3:**
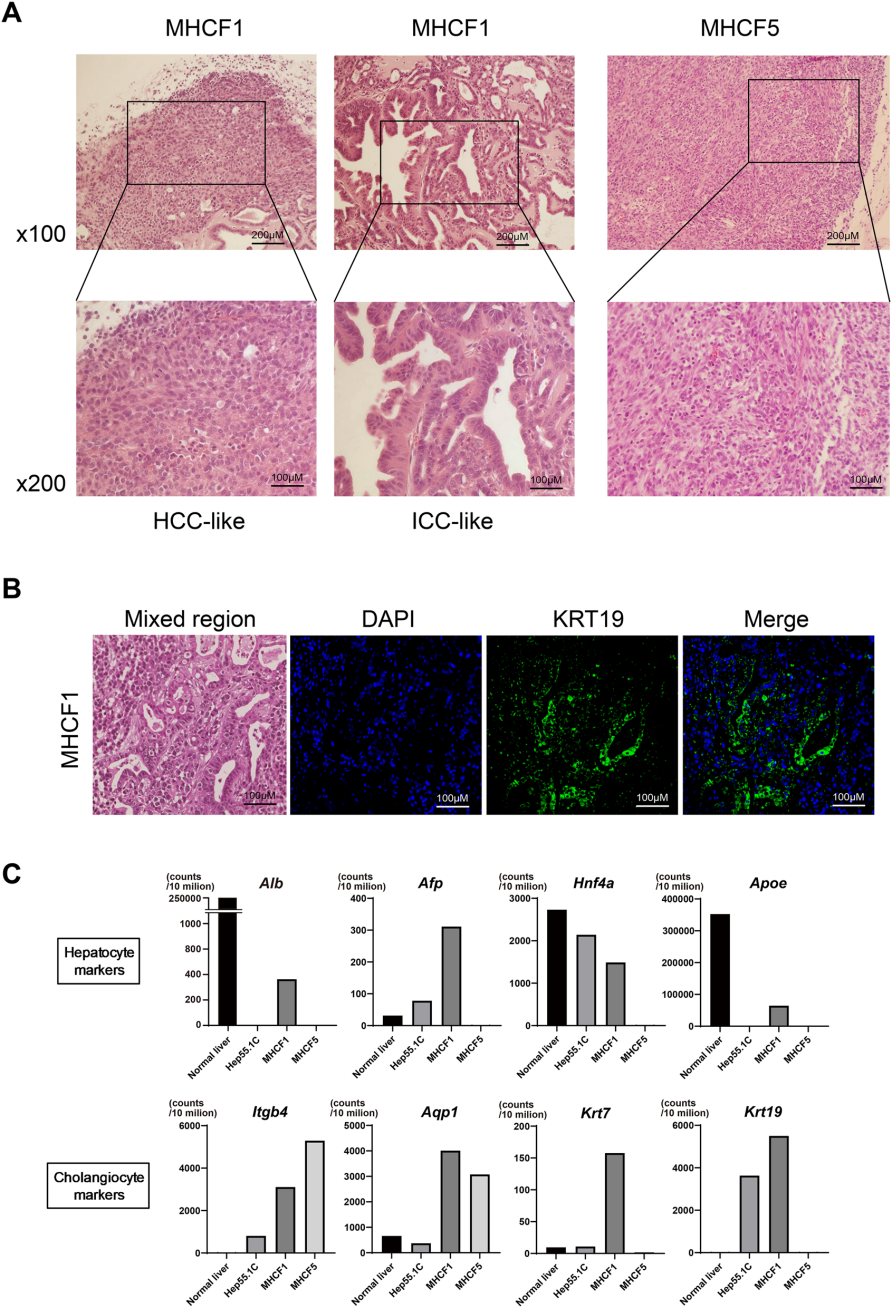
Histological findings and hepatocyte and cholangiocyte markers of the developed MHCF1- and MHCF5-derived tumors. (**A**) Hematoxylin and eosin staining of MHCF1- and MHCF5-derived tumors. (**B**) Mixed lesion of HCC and CCC in an MHCF1-derived tumor and immunofluorescent staining of KRT19. (**C**) RNA-seq results for hepatocyte and cholangiocyte markers in healthy liver, Hep55.1C cells, MHCF1 cells, and MHCF5 cells.

The histopathological features of MHCF1- and MHCF5-derived tumors were analyzed. MHCF1-derived tumors were composed of two cell components, namely, an HCC-like lesion and an intracellular cholangiocarcinoma-like lesion (Fig. 3A). Therefore, MHCF1-derived tumors were considered to be combined HCC and CCC. KRT19 expression was confirmed in the intracellular cholangiocarcinoma-like lesions by immunohistochemical staining (Fig. 3B). In contrast, MHCF5-derived tumors were composed of mesenchymal and fibrotic cells, as reported for sarcomatous HCC after repeated anti-cancer treatment^9^.

We performed RNA-seq analysis of MHCF1 and MHCF5 cells together with healthy liver and Hep55.1C cells, which were derived from carcinogen-induced liver tumors developed in C57BL/6 mice^10^. MHCF1 cells expressed hepatocyte markers such as *Alb*, *Afp*, hepatocyte nuclear factor 4 alpha (*Hnf4a*) and apolipoprotein e (*Apoe*) as well as cholangiocyte markers, such as integrin beta-4 (*Itgb4*), aquaporin 1 (*Aqp1*), keratin 7 (*Krt7*), and *Krt19.* In contrast, MHCF5 cells lost the expression of hepatocyte markers, but maintained the expression of cholangiocyte markers (*Itgb4, Aqp1*, and *Krt7*) (Fig. 3C). Hepatocyte markers were expressed at a higher level in MHCF1 cells than in Hep55.1C cells, while Hep55.1C cells expressed cholangiocyte markers to some degree. These results were confirmed by RTD-PCR (data not shown).

### Establishment of orthotopic liver tumor models of MHCF1 and MHCF5 cells by splenic tumor cell injection

**Figure 4:**
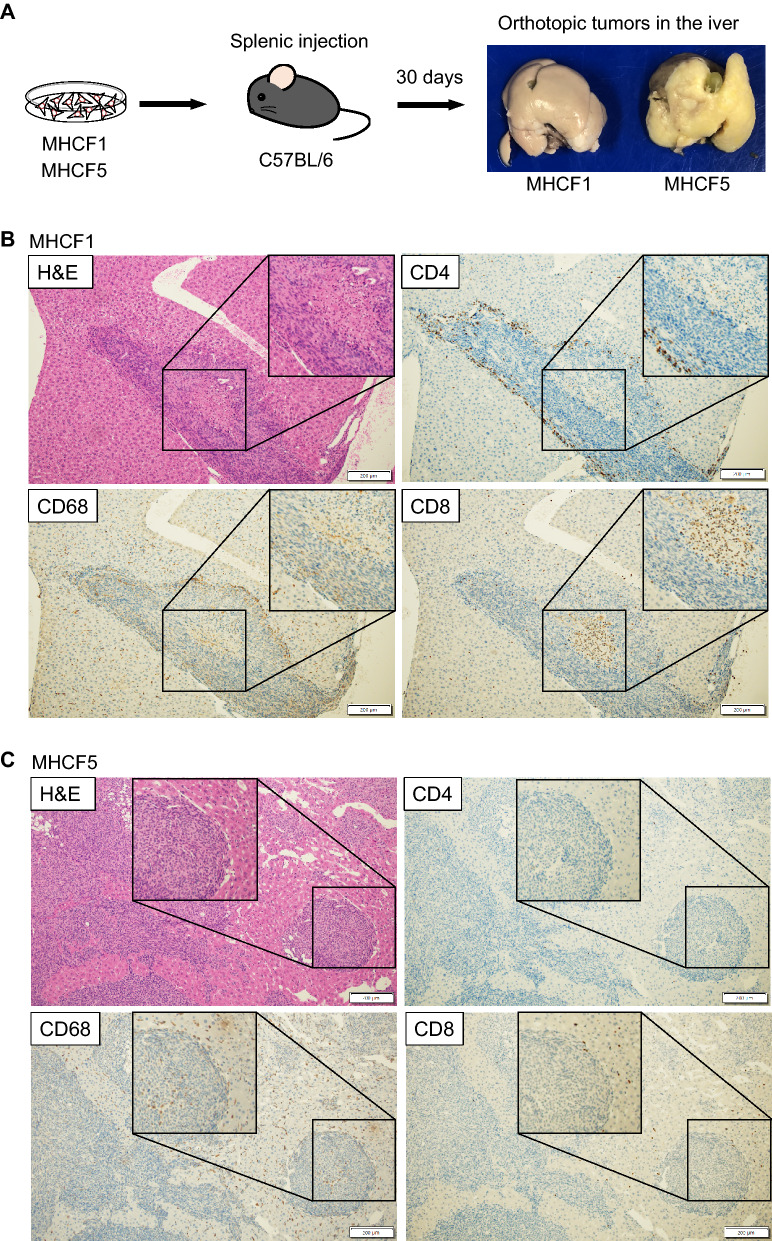
Establishment of orthotopic liver tumor models of MHCF1 and MHCF5 cells by splenic tumor cell injection. (**A**) Experimental procedure and development of orthotopic liver tumors of MHCF1 and MHCF5 cells. (**B**) Hematoxylin and eosin staining of a liver section, and immunohistochemical staining of immune cells in an orthotopic tumor derived from MHCF1 cells. (**C**) Hematoxylin and eosin staining of a liver section, and immunohistochemical staining of immune cells in an orthotopic tumor derived from MHCF5 cells.

To examine whether MHCF1 and MHCF5 cells could develop into tumors in the liver, they were injected into the spleen of C57BL/6 mice. At 30 days after injection, the mice were sacrificed and the livers were examined. We found that the whole liver was almost completely occupied with a tumor (white colored lesion) (Fig. 4A). The infiltration of immune cells was investigated by immunohistochemical staining of CD4-, CD8-, and CD68-positive cells. Interestingly, CD4 T cells preferentially accumulated in the marginal area of MHCF1-derived tumors, while CD8 T cells infiltrated the MHCF1-derived tumors (Fig. 4B). In contrast, no CD4 or CD8 T cell accumulation was observed in MHCF5-derived tumors (Fig. 4C). For CD68-positive monocytes/macrophages, there was no difference in the degree of infiltration between MHCF1- and MHCF5-derived tumors.

### Gene expression profiling of MHCF1 and MHCF5 cells

To reveal the molecular signatures of MHCF1- and MHCF5-derived tumors, gene expression profiling was performed using a gene chip. The gene expression profiles of MHCF1 cells, MHCF5 cells, and healthy mouse liver were compared. First, we examined the expression of highly expressed genes (57 representative genes that were within the top 1.5% of all transcripts) in primary human hepatocytes (deduced from the personal RNA-sequencing data of PXB cells; PhoenixBio, Hiroshima, Japan). In addition to *Alb* and *Hnf4a,* other hepatocyte-specific genes such as apolipoproteins (*Apoe*, *Apob*, and *Apoa1*), coagulation factors (*F5* and *Fgb*), bile acid transporter (*Abcc3*), and complement proteins (*C2* and *C3*) were expressed in MHCF1 cells, but not in MHCF5 cells (Supplemental Fig. 3).

**Figure 5:**
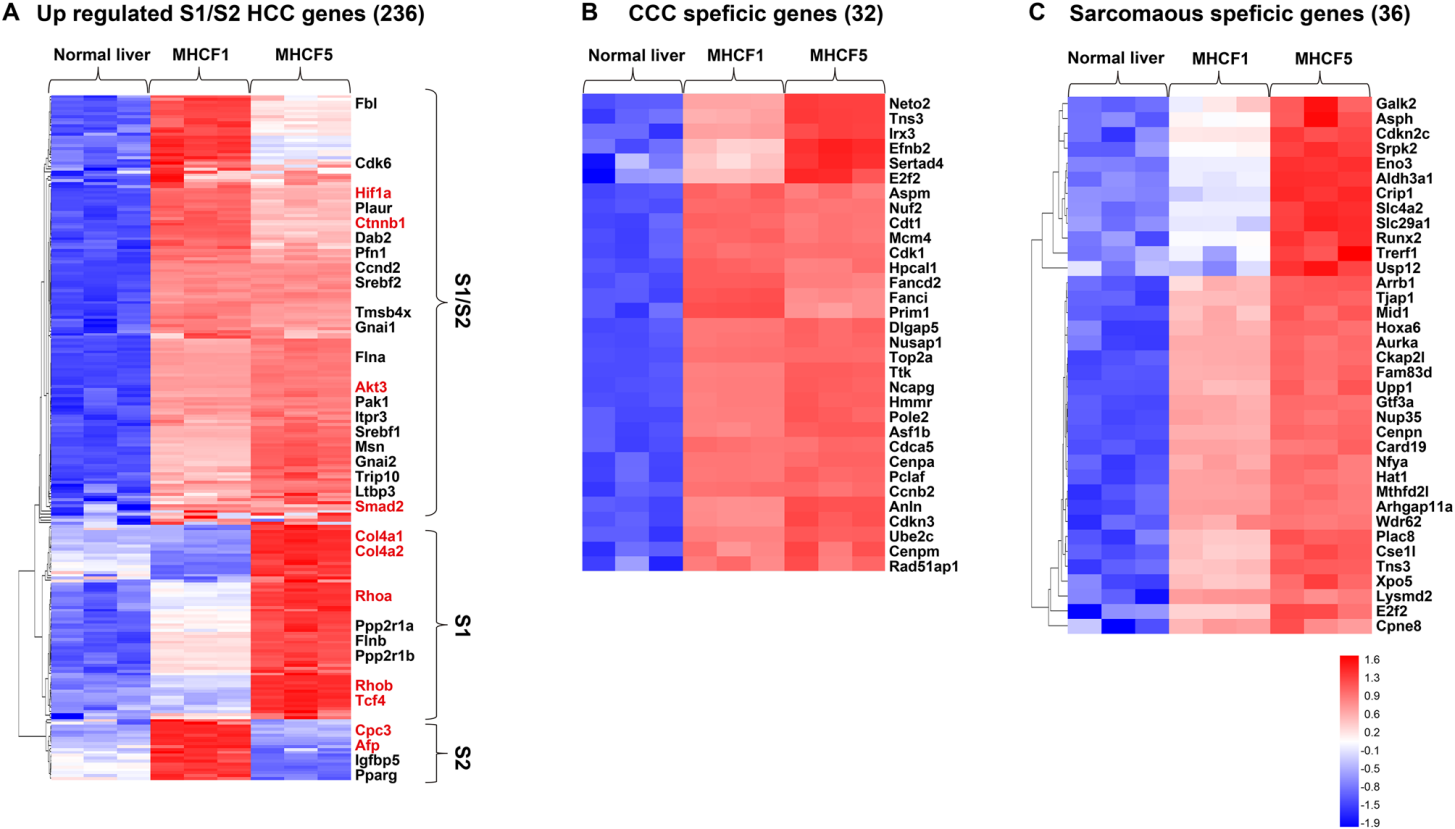
One-way hierarchical clustering of MHCF1 cells, MHCF5 cells, and healthy liver. (**A**) One-way hierarchical clustering of MHCF1 cells, MHCF5 cells, and healthy liver using 236 upregulated S1/S2 HCC genes. (**B**) One-way hierarchical clustering of MHCF1 cells, MHCF5 cells, and healthy liver using 32 CCC-specific genes. (**C**) One-way hierarchical clustering of MHCF1 cells, MHCF5 cells, and healthy liver using 36 sarcomatous-specific genes.

We next examined the expression of HCC-specific genes in MHCF1 and MHCF5 cells according to Hoshida’s HCC classification (S1, S2, and S3)^11^. In the GeneChip Mouse Gene 1.0 ST Array (Affymetrix, Santa Clara, CA) used in this study, 199 S1 genes, 101 S2 genes, and 208 S3 genes could be analyzed. We found that 79% of S1 and S2 genes (236 out of 300) were upregulated in MHCF1 and/or MHCF5 cells (Fig. 5A), while 23% of S3 genes (48 out of 208) were upregulated in MHCF1 and/or MHCF5 cells. Thus, MHCF1 and MHCF5 cells shared the features of S1 and S2 subclasses rather than the S3 subclass. Among the 236 upregulated genes in MHCF1 and/or MHCF5 cells, 149 genes were commonly upregulated in both cell lines, including *Akt3*, *Ctnnb1*, *Smad2*, and *Hif1a,* indicating the activation of *Wnt*, *Tgf-β*, *Akt*, and *Myc* signaling. Sixty-seven genes were upregulated only in MHCF5 cells, including *Col4*, *Rho*, and *Tcf4*, indicating the activation of *Wnt* and *Tgf-β* signaling. Twenty-one genes were upregulated only in MHCF1 cells, including the tumor markers *Afp* and *Cpc3*. The results indicated that MHCF1 cells more resembled the S2 subclass, while MHCF5 cells more resembled the S1 subclass.

Next, on the basis of the previously reported gene expression data of healthy biliary epithelial cells and CCC cells (GSE22633)^12^, we selected 32 CCC-specific genes and examined their expression in MHCF1 and MHCF5 cells. These CCC-specific genes were expressed in MHCF1 and MHCF5 cells (Fig. 5B). Similarly, we selected 32 sarcomatous-specific genes deduced from CCC with sarcomatous changes^12^ and examined their expression in MHCF1 and MHCF5 cells. Sarcomatous-specific genes were expressed at a higher level in MHCF5 cells than in MHCF1 cells (Fig. 5C). These results supported the histological findings of MHCF1- and MHCF5-derived tumors; MHCF1-derived tumors consisted of a combined type of HCC sharing the features of HCC and CCC, whereas MHCF5-derived tumors were poorly differentiated CCC with sarcomatous features.

Finally, we performed pathway comparisons among MHCF1 cells, MHCF5 cells, and healthy liver as previously described^13^. Compared with healthy liver, the expression of cell cycle (*Cdk*, etc.), DNA damage response (*Brca*, *Rb*, *p53*, etc.), *Erad* pathway (endoplasmic reticulum-associated), and oncogene (sonic hedgehog [*Shh*] and *Src*) genes was upregulated in MHCF1 and MHCF5 cells, whereas the expression of coagulation, complement, lectin, and nuclear receptor genes was downregulated (Table 1) (Supplemental Fig. 4A). Interestingly, when comparing MHCF1 and MHCF5 cells, the expression of *Tgf-β* and inflammation pathway genes was upregulated in MHCF5 cells compared with MHCF1 cells, and the expression of coagulation genes was downregulated in MHCF5 cells compared with MHCF1 cells (Table 1) (Supplemental Fig. 3B). Therefore, MHCF5 cells expressed genes that are characteristic of epithelial to mesenchymal transition.

**Table 1:** Commonly and differentially disregulated signaling pathways based on the gene expression profiles of MHCF1 and MHCF5 cells.

No	Biocarta pathway	Pathway description	Number of genes	LS permutation *p* value	KS permutation *p* value	Upregulated (normal vs. MHCF1/5)
**Commonly disregulated signaling pathways based on the gene expression profiles of MHCF1 and MHCF5 cells**						
1	m_atrbrcaPathway	Role of BRCA1, BRCA2 and ATR in cancer susceptibility	19	0.00001	0.00396	MHCF1/5
2	m_cellcyclePathway	Cyclins and cell cycle regulation	27	0.00001	0.00276	MHCF1/5
3	m_rbPathway	RB tumor suppressor/checkpoint signaling in response to DNA damage	13	0.00001	0.0062	MHCF1/5
4	m_extrinsicPathway	Extrinsic prothrombin activation pathway	14	0.00002	0.01748	Normal
5	m_p53Pathway	p53 signaling pathway	17	0.00025	0.00044	MHCF1/5
6	m_eradPathway	ER-associated degradation (ERAD) pathway	17	0.00026	0.03851	MHCF1/5
7	m_cdc25Pathway	Cdc25 and Chk1 regulatory pathway in response to DNA damage	9	0.00033	0.03746	MHCF1/5
8	m_classicPathway	Classic complement pathway	8	0.00067	0.00771	Normal
9	m_EfpPathway	Estrogen-responsive protein Efp controls cell cycle and breast tumors growth	15	0.00082	0.01525	MHCF1/5
10	m_ptc1Pathway	Sonic hedgehog (SHH) receptor Ptc1 regulates cell cycle	12	0.00158	0.09869	MHCF1/5
11	m_lectinPathway	Lectin-induced complement pathway	6	0.00197	0.00626	Normal
12	m_badPathway	Regulation of BAD phosphorylation	23	0.00505	0.13066	MHCF1/5
13	m_srcRPTPPathway	Activation of Src by protein-tyrosine phosphatase alpha	10	0.0073	0.27975	MHCF1/5
14	m_nuclearRsPathway	Nuclear receptors in lipid metabolism and toxicity	24	0.04443	0.15073	Normal
15	m_tcrPathway	T-cell receptor signaling pathway	39	0.08731	0.00055	MHCF1/5

No	Biocarta pathway	Pathway description	Number of genes	LS permutation *p* value	KS permutation *p* value	Upregulated (MHCF1 vs. MHCF5)
**Differentially disregulated signaling pathways based on the gene expression profiles of MHCF1 and MHCF5 cells**						
1	m_extrinsicPathway	Extrinsic prothrombin activation pathway	14	0.00016	0.00366	MHCF1
2	m_mta3Pathway	Downregulated of MTA-3 in ER-negative breast tumors	23	0.00091	0.00134	MHCF5
3	m_tgfbPathway	TGF-beta signaling pathway	18	0.00182	0.09829	MHCF5
4	m_classicPathway	Classic complement pathway	8	0.00192	0.43013	MHCF1
5	m_eicosanoidPathway	Eicosanoid metabolism	17	0.00291	0.12272	MHCF5
6	m_ndkDynaminPathway	Endocytotic role of NDK, phosphins, and dynamin	14	0.00691	0.37843	MHCF5
7	m_tob1Pathway	Role of Tob in T-cell activation	14	0.01064	0.39454	MHCF5
8	m_lectinPathway	Lectin-induced complement pathway	6	0.01682	0.63191	MHCF1
9	m_DNAfragmentPathway	Apoptotic DNA fragmentation and tissue homeostasis	44	0.02202	0.00001	MHCF5
10	m_hdacPathway	Control of skeletal myogenesis by HDAC	26	0.02903	0.44623	MHCF5
11	m_eea1Pathway	The role of FYVE-finger proteins in vesicle transport	5	0.03142	0.7613	MHCF1
12	m_inflamPathway	Cytokines and inflammatory response	13	0.03988	0.44185	MHCF5
13	m_nktPathway	Selective expression of chemokine receptors during T-cell polarization	16	0.06403	0.0176	MHCF5
14	m_pcafpathway	Information-processing pathway at the IFN-beta enhancer	42	0.14201	0.00001	MHCF5

### Whole-exon sequencing of MHCF1 and MHCF5 cells

To examine the somatic mutations of MHCF1 and MHCF5 cells, whole-exon sequencing was performed and compared with healthy mouse liver (C57BL/6 mice). A total of 477 genes were mutated in MHCF1 cells and 464 genes were mutated in MHCF5 cells. MHCF1 and MHCF5 cells shared 371 common mutated genes, and gene networks consisting of these genes were analyzed by MetaCore Bioinformatics software (Thomson Reuters; https://portal.genego.com/) (Table 2). Tumor suppressor (*Tp53bp1*), stem cell (*Sox9*), oncogene (*Braf*), growth factor (*Pdgfa*), cell cycle (*Bub1*), and Notch (*Dll4*)-related genes were mutated. Among the 477 mutated genes, 106 genes were unique to MHCF1 cells. The MHCF1 unique mutations consisted of protein misfolding (*Hsp*-related genes) and acetylcholine receptor (*Chrnb4* and *Chrm5*) genes that possibly act on cancer cell processes, and tumor suppressor (*Adamts1* and *Adamts5*) and cell cycle (*Aurkb* and *Bub1*) genes (Table 3). In contrast, 93 genes were unique to MHCF5 cells. MHCF5 unique mutations consisted of hedgehog signaling (*Csnk1a1, Foxa2, Disp1, Phox2b, Lrp2, Sox9*, and *Zeb1*) and *Wnt* signaling (*Lrp5*, *Ccne1*, and *Pax6*) genes (Table 3). Thus, MHCF1 and MHCF5 cells shared common cancer-related gene mutations, and MHCF5 cells obtained a cancer stem cell-like signature of *Shh* and *Wnt* signaling.

**Table 2:** Common exon mutations in MHCF1 and MHCF5 cells and their related signaling networks.

*P* value	Networks	Mutated genes
0.009	Neurophysiological process_Olfactory transduction	OR4F3, 4, 5, 16, 17, 29, OR5AS1, OR5D14, OR5L2, OR5M1, 3, 9, 10, 11,
		OR8H1, 2, OR8I2, OR8J3, OR8K1, 5, OR9G1, 4, OR5L1
0.01	DNA damage_Core	TP53BP1, RFC1
0.013	Development_Cartilage development	SOX9, BMPR2, FBN1,
0.018	Development_Regulation of telomere length	HSP90AB1
0.018	Transport_Bile acid transport and its regulation	ABCB11, SULT2A1
0.019	Signal transduction_Androgen receptor signaling cross-talk	BRAF, PAK6
0.019	Inflammation_Complement system	SERPING1, CD59
0.019	Cell adhesion_Integrin priming	CXCR4, PLCB2
0.02	Apoptosis_Anti-apoptosis mediated by external signals via NF-κB	PDGFRA, ADCY5
0.02	Apoptosis_Apoptotic mitochondria	SIVA1, AVEN, HSPA1A, HSPA1B, HSPA2
0.02	Translation_Selenium pathway	SELENOI, SEPSECS, SRY
0.021	Cell adhesion_Platelet-endothelium-leucocyte interactions	SERPING1, SIRPA, THBS1, APOB, PDGFRA
0.022	Transcription_Chromatin modification	EPC1, MORF4L2, BRCA2, ARID1A, SIRT5
0.023	Cell cycle_G2-M	BUB1, BUB1B, AURKB, ESCO1, BRCA2
0.024	Response to hypoxia and oxidative stress	PRDX3, EGLN3, CAT
0.025	Proliferation_Negative regulation of cell proliferation	CCNE1, ADAMTS1, IGFBP7
0.028	Immune response_T helper cell differentiation	TLR1, TIRAP, NFATC3
0.03	Transport_Calcium transport	CATSPER2, TRPV6, GPRC6A, CHRNB4, SLC24A5
0.032	Transcription_Transcription by RNA polymerase II	TAF4B, ELL3
0.035	Inflammation_TREM1 signaling	TIRAP, NFAT5, NFATC3
0.038	Signal transduction_NOTCH signaling	DLL4, FRZB, PDGFRA, BRAF
0.038	Cytoskeleton_Regulation of cytoskeleton rearrangement	ADRA2B, CHRM5, HTR1F

**Table 3:** Differential exon mutations between MHCF1 and MHCF2 cells and related networks.

*P* value	MHCF1 dominant networks	Mutated genes
**MHCF1 dominant exon mutations and related networks**		
0.0015	Protein folding_Response to unfolded proteins	HSPA1A, HSPA1B, HSP90AB1, HSPA2, UBOX5
0.0030	Muscle contraction	THBS1, DTNA, CHRNB4, TTN, CHRM5, CXCR4, MAP1A, CAPN3, MYH4
0.0130	Cell adhesion_Cell-matrix interactions	ECM1, TINAG, THBS1, ADAMTS5, ADAMTS1, FBN1
0.0291	Cell cycle_Mitosis	MACF1, AURKB, ASPM, BUB1, BUB1B, USP16, KNL1

*P* value	MHCF5 dominant networks	Mutated genes
**MHCF5 dominant exon mutations and related networks**		
0.0067	Development_Hedgehog signaling	CSNK1A1, FOXA2, DISP1, PHOX2B, PAX6, LRP2, ADCY5, HSP90AB1, SOX9, BMPR2, ZEB1
0.0030	Signal transduction_WNT signaling	LRP5, CCNE1, PAX6, FOXA2, ADCY5, PLCB2, BRAF, CSNK1A1, NFAT5, NFATC3
0.0055	Reproduction_GnRH signaling pathway	GRIP1, GRM8, BRAF, PLCB2, GABRR1, GABRR2

### Different sensitivity of MHCF1- and MHCF5-derived tumor against an anti-PD1 antibody

**Figure 6:**
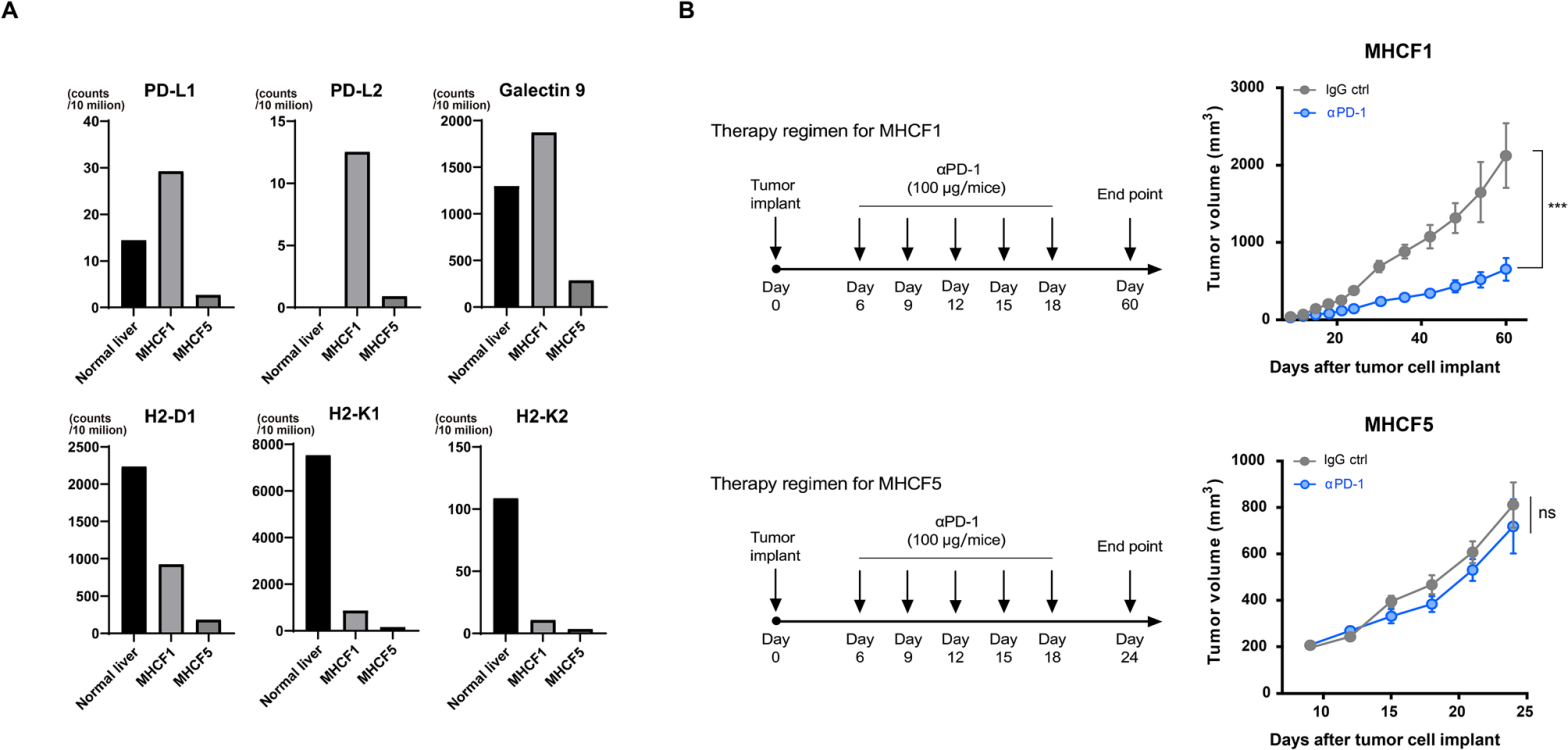
Different sensitivity of MHCF1- and MHCF5-derived tumors against the anti-PD1 antibody. (**A**) RNA-seq results for immune checkpoint molecules (PDL-1, PDL-2, and Galectin-9) and MHC class I molecules (H2-D1, H2-K1, and H2-K2) in MHCF1 and MHCF5 cells. (**B**) Anti-PD1 antibody (100 μg/mouse) was intraperitoneally administered every 3 days (total 5 times) to MHCF1- or MHCF5-derived tumor-bearing mice and tumor volumes were evaluated at 60 days and 25 days, respectively.

The sensitivity of MHCF1- and MHCF5-derived tumors against an anti-PD1 antibody was evaluated. The expression of immune checkpoint molecules (PDL-1, PDL-2, and Galectin-9) in MHCF1 and MHCF5 cells is shown in Fig. 6A. The expression of these immune checkpoint molecules was upregulated in MHCF1 cells compared to MHCF5 cells and healthy liver. For MHC class I molecules, the expression of H2-D1, H2-K1, and H2-K2 was downregulated in MHCF1 and MHCF5 cells compared to healthy liver.

An anti-PD1 antibody (100 μg/mouse for 5 times) was administered intraperitoneally to MHCF1- or MHCF5-derived tumor-bearing mice and tumor volumes were evaluated (Fig. 5). Interestingly, the anti-PD1 antibody reduced the volume of MHCF1-derived tumors by approximately 70%, while MHCF5-derived tumors showed no response to the anti-PD1 antibody. Thus, MHCF1- and MHCF5-derived tumors showed different sensitivity to the anti-PD1 antibody.

## Discussion

Treatment using ICIs for liver cancer has yielded some encouraging results, but the percentage of patients responding to single-agent therapies remains low. Therefore, the development of combination therapies for liver cancer is crucial and urgent. A syngeneic mouse model bearing an orthotopic or subcutaneous transplanted tumor is indispensable for the evaluation of the efficacy of ICIs or combinations of ICIs and other anti-tumor drugs. However, few syngeneic models of liver cancer are available. BNL-1MEA is a BALB/c-derived and chemically (methylcholanthrene epoxide) transformed cell line^14^. The HCA-1 cell line is derived from a spontaneously developed liver cancer in C3H mice^15,16^. However, genetically engineered mice such as Tg or knockout mice are made mainly from C57BL/6 mice; therefore, a syngeneic tumor model based on C57BL/6 mice is more useful to reveal the molecular mechanisms of drug resistance and immune-evading mechanisms of tumors in the setting of tumor microenvironments. The responsible genes for immune evasion by tumors can be analyzed by transplanting a tumor into responsible gene knockout or Tg mice. The RIL-175 cell line was established from C57BL/6 mice; however, this cell line has already been genetically modified by knocking out p53 (p53^−/−^) and transduced with H-RasV12^17^. The Hepa1-6 cell line is derived from C57L mice; however, we observed that subcutaneous Hepa1-6 HCC cells grew transiently in C57BL/6 mice and subsequently diminished^18^. The Hep55.1C cell line is derived from carcinogen-induced liver tumors of C57BL/6 mice^10^ and utilized in a syngeneic orthotopic murine model of HCC^19^. However, the etiology of liver cancer in this model is different from that of the clinical setting.

In this study, we developed new mouse liver tumor cell lines (MHCF1 and MHCF5 cells) from HCV-Tg mice fed an Ath + HFD. We previously reported an Ath + HFD mouse model that develops hepatic steatosis, inflammation, fibrosis, and insulin resistance, resembling human non-alcoholic steatohepatitis^20^. Non-alcoholic fatty liver disease and non-alcoholic steatohepatitis are frequently associated with chronic hepatitis C and they are accelerated in HCV-related liver disease^21^. Therefore, tumor cell lines derived from HCV-Tg mice fed an Ath + HFD would have clinical significance.

Our cell lines were successfully transplanted into the subcutaneous space of syngeneic C57BL/6 mice and tumor growth was evaluated. In addition, these cell lines efficiently developed orthotopic tumors in the liver of syngeneic C57BL/6 mice. These cell lines are unique, because no host tumor suppressor genes or oncogenes were manipulated. A trace mount of HCV-RNA was detected in both cell lines; however, no HCV protein was detected. Thus, the effect of HCV proteins on the tumor phenotypes of these cell lines would be minimal.

Histologically, MHCF1 tumors showed the combined type of HCC sharing the features of epithelial-like HCC lesions and ductal cholangiocyte-like lesions. In contrast, MHCF5-derived tumors showed the features of poorly differentiated sarcomatous HCC.

According to Hoshida’s HCC classification (S1, S2, and S3)^11^, MHCF1 and MHCF5 cells were classified as S1 and/or S2 subclasses, which are associated with poor clinical survival. MHCF1 was more like the S2 subclass with the activation of *Akt* and *Myc* and positivity for the tumor marker *Afp*. In contrast, MHCF5 was more like the S1 subclass with the activation of *Wnt* and *Tgf-β* (Fig. 5A). It could be speculated that MHCF1 cells originated from progenitor cells that can develop into hepatocytes and cholangiocytes. We performed 3 cycles of transplantation of MHCF1 cells into NOD-SCID mice. During these repeated transplantation steps, rapidly growing and aggressive tumors could be selected in vivo. Interestingly, the in vivo selection of MHCF1 cells enabled us to isolate tumor cells (MHCF5 cells) with a different phenotype and genotype compared with the original MHCF1 cells.

Gene expression profiling and whole-exon sequencing supported the histological findings of the MHCF1- and MHCF5-derived tumors. Interestingly, MHCF5 cells lost the hepatocyte gene signature, but retained the expression of representative CCC marker genes together with a sarcomatous gene signature (Fig. 5B,C). It is characteristic that MHCF5 cells obtained an epithelial to mesenchymal transition signature and activated the cancer stem cell-like signature of *Shh* and *Wnt* signaling.

Interestingly, the MHCF1- and MHCF5-derived tumors showed different sensitivity to the anti-PD1 antibody. The anti-PD1 antibody reduced the volume of MHCF1-derived tumors by 70%, while MHCF5-derived tumors were resistant to this antibody (Fig. 6B). Interestingly, CD8 T cells infiltrated the MHCF1-derived tumors, but no CD8 T cells were found within the MHCF5-derived tumors (Fig. 4). Although we could not see any difference in the infiltration of CD68-positive cells between these tumors, further studies evaluating myeloid-derived suppressor cells should be performed. In this study, the expression of immune checkpoint molecules, such as PD-L1, PD-L2, and Galectin-9, was higher in MHCF1 cells than in MHCF5 cells (Fig. 6A), which might be related to the favorable response of MHCF1 cells to the anti-PD1 antibody to some degree. However, the expression of these molecules is regulated by multiple factors including MAPK, PI3K, AKT HIF1, STAT3, and NF-κB^22^. Moreover, a recent report showed that the activation of *Wnt* signaling could be related to resistance against anti-PD1 therapy^23^. *Shh* and *Wnt* signaling are co-operating signaling pathways that are essential for embryonic development^24^. The detailed underlying mechanisms of the different responses of MHCF1 and MHCF5 cells to the anti-PD1 antibody should be studied further.

In summary, we newly developed two mouse liver tumor cell lines, MHCF1 and MHCF5 cells, derived from HCV-Tg/Ath + HFD mice. MHCF1-derived tumors consisted of a combined type of HCC, whereas MHCF5-derived tumors were CCC with a malignant phenotype and resistant to anti-PD1 therapy. These cell lines could be useful for the generation of syngeneic mouse tumor transplant models and the identification of new biomarkers and molecular mechanisms of ICI resistance.

## Materials and methods

### Mice

The generation and characterization of Ath + HFD mice were performed as previously described^20^. HCV-Tg mice were generated as previously described^8^. Pdgfc-Tg mice were kindly provided by Dr. Jean S. Campbell (University of Washington, Seattle, WA)^7^. C57BL/6 J and NOD.CB17-Prkdcscid/J mice were purchased from Charles River Laboratories Japan (Yokohama, Japan). Colonies of HCV-Tg and Pdgfc-Tg mice were maintained by crossing with C57BL/6 J mice for at least 5 generations. All animal experiments were approved by the Ethics Committee for the Care and Use of Laboratory Animals at the Takara-Machi Campus of Kanazawa University, Japan and were carried out in compliance with the ARRIVE guidelines 2.0. All experiments were performed in accordance with relevant guidelines and regulations.

### Cell culture

The cells were cultured in Dulbecco’s modified Eagle’s medium (Life Technologies, Carlsbad, CA) containing 10% fetal bovine serum, 100 U/mL penicillin, and 100 mg/mL streptomycin and maintained at 37 °C with 5% CO_2_. Culture plates were coated with Collagen Solution (STEMCELL Technologies, Inc., Vancouver, Canada).

### Genotyping PCR

Total DNA was isolated using a DNeasy Blood & Tissue Kit (Qiagen, Valencia, CA). PCR was performed using the GoTaq Green Master Mix (Promega, Madison, WI) according to the manufacturer’s instructions. The primers used for the detection of *Pdgfc* were forward 5′-CATACTTATCCAAGAAATACGGTC-3′ and reverse 5′-CTCTCGGTTCAAGATATCGAA-3′, and for the detection of HCV core were forward 5′-CAACCCTACGTACAGCTG-3′ and reverse 5′-GGTAGTCAACCATGCACC-3′.

### Cell viability

The cells were seeded in 96-well plates at a density of 5,000 cells/well. At 24, 48, and 72 h after incubation, 10 μL of Cell Counting Kit-8 solution (Dojindo Molecular Technologies, Inc., Rockville, MD) was added to each well. The plates were incubated for 1 h at 37 °C, and absorbance was measured at 450 nm using a microplate reader (Sunrise Tecan, Mannedorf, Switzerland).

### Generation of MHCF1 and MHCF5 cell lines

Liver tumors derived from HCV-Tg/Ath + HFD mice were dissociated with a Tumor Dissociation Kit, Mouse (Miltenyi Biotec, Bergisch Gladbach, Germany) according to the manufacturer’s instructions. The cells were seeded on collagen-coated dishes and grown and passaged several times. The established cell lines were transplanted subcutaneously into immune-deficient NOD-SCID mice. The tumor cells were dissociated and grown on dishes. We named this cell line MHCF1. MHCF1 cells were repeatedly transplanted into NOD-SCID mice, and the developed tumors were dissociated and passaged on culture dishes several times. We performed these procedures three times. After three cycles, the obtained cells were seeded on 96-well plates with limiting dilution and 12 clones were obtained. Six of the 12 clones were transplanted subcutaneously into syngeneic C57BL/6 mice and all clones developed into tumors. We selected one fast growing clone and named it MHCF5.

### Transplantation and mouse experiments

Cells (5.0 × 10^5^ cells in 100 μL phosphate-buffered saline) were mixed with 100 μL Matrigel Matrix Basement Membrane High Concentration (Corning, Inc., Corning, NY). The cells were injected subcutaneously into 8-week-old male NOD.CB17-Prkdcscid/J or C57BL/6 mice. For the establishment of orthotopic liver tumor models, the cells (1.0 × 10^6^ cells in 100 μL phosphate-buffered saline) were injected via the spleen into 8-week-old male C57BL/6 mice. At 30 days after injection, hepatic tumors were evaluated by hematoxylin and eosin staining and immunohistochemistry.

Purified anti-mouse PD1 monoclonal antibody, *InVivo*MAb anti-mouse PD-1 (CD279) (catalog no. BE0146, clone: RMP1-14), and control Ig, *InVivo*MAb rat IgG2a isotype control (catalog no. BE0089, clone: 2A3), were purchased from BioXCell (Lebanon, NH). The anti-PD1 antibody (100 μg/mouse) was intraperitoneally administered to MHCF1- or MHCF5-derived tumor-bearing mice every 3 days from day 6 to 18 (total 5 times), and tumor volumes were evaluated until 60 days and 25 days, respectively.

### Tumor cell dissociation from transplanted mice

A Tumor Dissociation Kit, Mouse (Miltenyi Biotec) was used according to the manufacturer’s instructions. Briefly, tumors were cut into small pieces in Petri dishes and transferred into a gentleMACS C Tube containing enzyme mix. The tissue was dissociated using a gentleMACS Dissociator (Miltenyi Biotec). The cell suspensions were washed three times with phosphate-buffered saline and passed through a 100-μm strainer with 10 mL Dulbecco’s modified Eagle’s medium. These cells were cultured as described above.

### Immunohistochemistry and immunofluorescence staining

Formalin-fixed paraffin-embedded tissue sections, 4 μm in thickness, were deparaffinized with Histo-Clear. For antigen retrieval, the tissue samples were autoclaved in 0.01 M citrate buffer or Tris–EDTA (pH 9.0) for 5 min at 121 °C. Endogenous peroxidase was inactivated by Peroxidase-Blocking Solution (DAKO, Tokyo, Japan). The samples were blocked with blocking buffer (DAKO, Tokyo, Japan), and incubated at 4 °C overnight with primary antibodies against CD4 (rabbit monoclonal, catalog no. ab183685; Abcam, Cambridge, MA), CD8 (rabbit polyclonal, catalog no. ab203035; Abcam), and CD68 (rabbit polyclonal, catalog no. ab125212; Abcam). Sections were stained by DAB chromogen with a Histofine Simple Stain MAX-PO(R) + Histofine SAB-PO(M) Kit (NICHIREI CORPORATION, Tokyo, Japan), and counterstained with hematoxylin.

For immunofluorescence staining, the slides were incubated with an Alexa Fluor 488-labeled anti-cytokeratin 19 antibody (1:200; Abcam) diluted in 1% bovine serum albumin/phosphate-buffered saline for 2 h at room temperature. The slides were mounted using DAPI, and the cells were viewed using an image analysis system (BIOREVO BZ-9000; Keyence, Osaka, Japan).

### Quantitative RTD-PCR

Total RNA was isolated using a High Pure RNA Isolation Kit (Roche Applied Science, Pleasanton, CA), and cDNA was synthesized with a High Capacity cDNA Reverse Transcription Kit (Applied Biosystems, Foster City, CA) according to the manufacturers’ instructions. RTD-PCR was performed using the 7900HT Real-Time PCR System (Applied Biosystems). The primer pairs and probes for mouse genes (*Alb, Afp, Hnf4a, Aqp1, Krt7, Krt19*, and *Gapdh*) were obtained from the TaqMan assay reagents library.

### RNA-seq and gene chip analysis

Total RNA was isolated using a High Pure RNA Isolation Kit (Roche Applied Science); 5 µg of total RNA was utilized for RNA amplification and synthesis of double-stranded cDNAs according to the TruSeq RNA Sample Prep guidelines (Illumina, San Diego, CA). The paired-end reads of each sample were aligned to the mouse genome (mm10) using Subread^25^, and transcript abundance was shown by the count data by using HTSeq^26^. Expression data were adjusted by a total of 10 million counts. Gene chip analysis of MHCF1 cells, MHCF5 cells, and mouse liver was performed using a GeneChip Mouse Gene 1.0 ST Array (Affymetrix) as described previously^27^. Functional ontology enrichment analysis was conducted to compare the BioCarta Pathway process distribution of the differentially expressed genes^13^. LS/KS permutation tests were performed for pathway comparison (*P* < 0.05) (BRB-ArrayTools; https://brb.nci.nih.gov/BRB-ArrayTools).

### Whole-exon sequencing

A whole-exon sequencing was performed as previously described^28^. A Sure-Select Human All Exon V4 Kit (Agilent Technologies, Santa Clara, CA) was used for whole-exon capture, and the HiSeq 2000 Sequencing System (Illumina) was used for massive parallel sequencing. Sequence reads were mapped against the UCSC Genome Browser mm10. Sequence variations, including single nucleotide polymorphisms and insertions/deletions, were detected using Genome Analysis Toolkit software (Broad Institute, Cambridge, MA). Whole-exon sequencing and analysis was performed at Riken Genesis (Tokyo, Japan). Pathway analysis was conducted using MetaCore (Thomson Reuters, New York, NY).

### Statistical analysis

For the animal experiments, a sample size of 5 was chosen for each experimental group. Data represent the mean ± standard error of the mean from two or three independent experiments. The significance of between-group comparisons was tested by one-way or two-way analysis of variance with Bonferroni’s multiple comparisons test using Prism 8.0 software (GraphPad Software, La Jolla, CA).

## Supplementary Information

Supplementary Information.

## Abbreviations

Ath + HFD: Atherogenic and high-fat diet
CCC: Cholangiocellular carcinoma
HCC: Hepatocellular carcinoma
HCV: Hepatitis C virus
ICIs: Immune checkpoint inhibitors
MHCF1: Mouse liver tumor cells derived from full-length hepatitis C virus transgenic no. 1
MHCF5: Mouse liver tumor cells derived from full-length hepatitis C virus transgenic no. 5
Pdgfc: Platelet-derived growth factor c
Tg: Transgenic
TKIs: Tyrosine kinase inhibitors
