# Supplementary material for: Establishment of liver tumor cell lines from atherogenic and high fat diet fed hepatitis C virus transgenic mice
Source: Sci Rep. 2021 Jun 22;11:13021. doi: 10.1038/s41598-021-92128-9 (PMC8219799; doi:10.1038/s41598-021-92128-9)
Supplement: Supplementary file 1 — Supplementary Information. [file 41598_2021_92128_MOESM1_ESM.docx]

**Establishment of liver tumor cell lines from atherogenic and high fat diet fed hepatitis C virus transgenic mice**

Takayoshi Shirasaki ^1,2*^, Kazuhisa Murai^1,2*^, Masao Honda^1,2^, Hikari Okada^1^, Yuika innami^2^, Atsumu Yamada^2^, Tetsuro Shimakami^1^, Kazunori Kawaguchi^1^, Taro Yamashita^1^, Yoshio Sakai^1^, and Shuichi Kaneko^1^

^1)^ Department of Gastroenterology, Kanazawa University Graduate School of Medicine, Kanazawa, Japan

^2)^ Department of Laboratory Medicine, Kanazawa University Graduate School of Health Medicine, Kanazawa, Japan


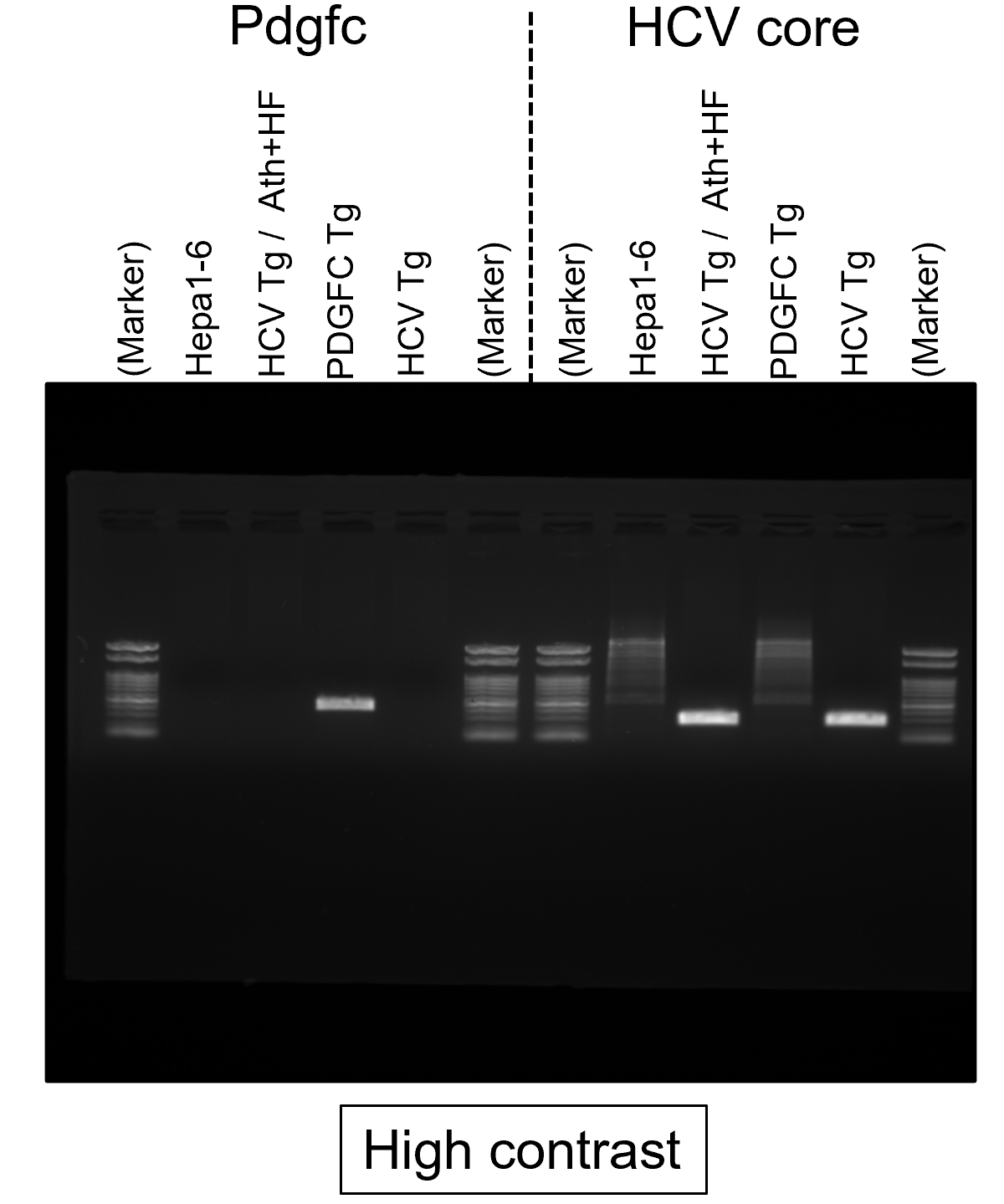

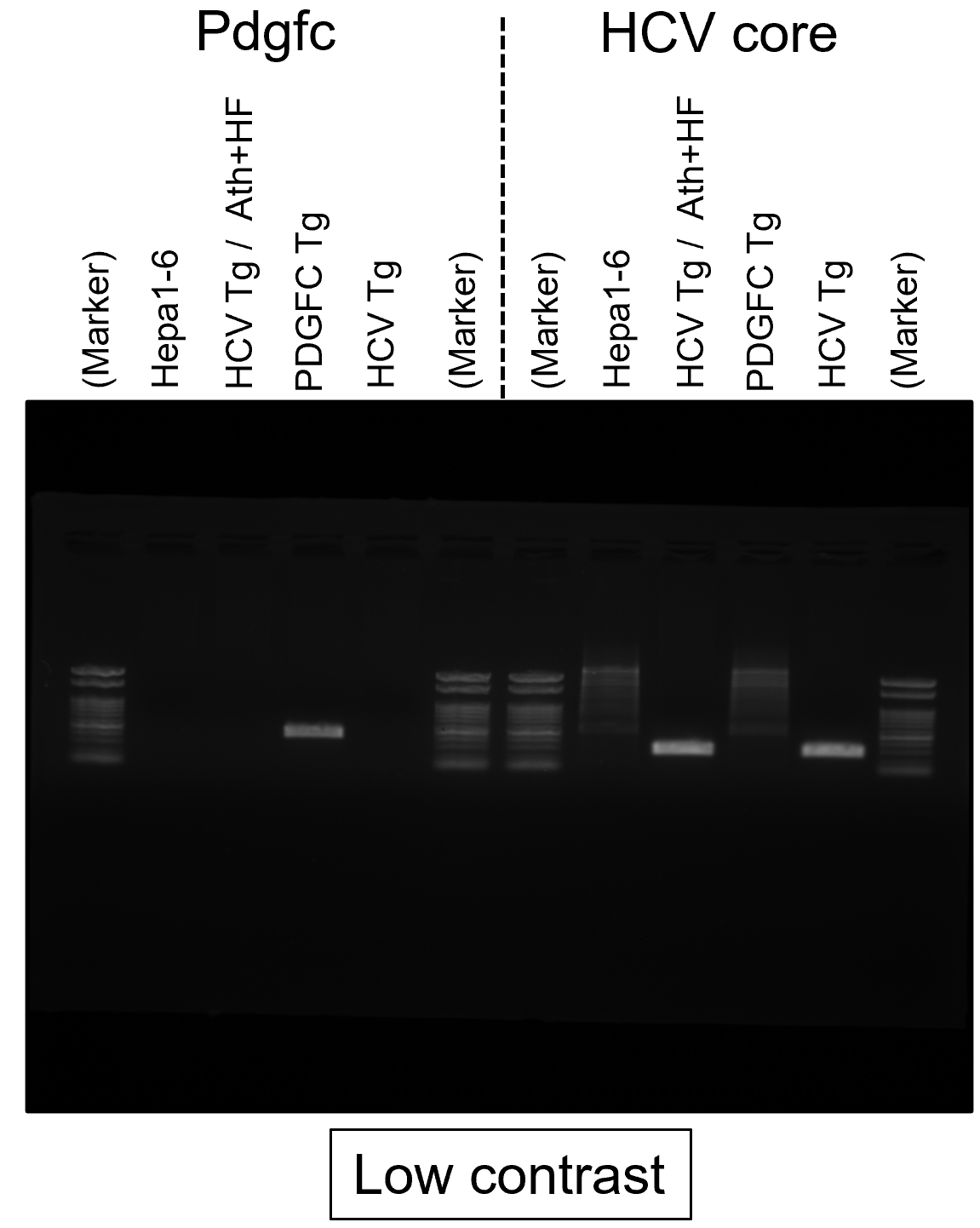


**Supplemental Figure 1.**

High- and low-contrast images of the original full-length agarose gels shown as cropped images in Figure 1C.

**B**


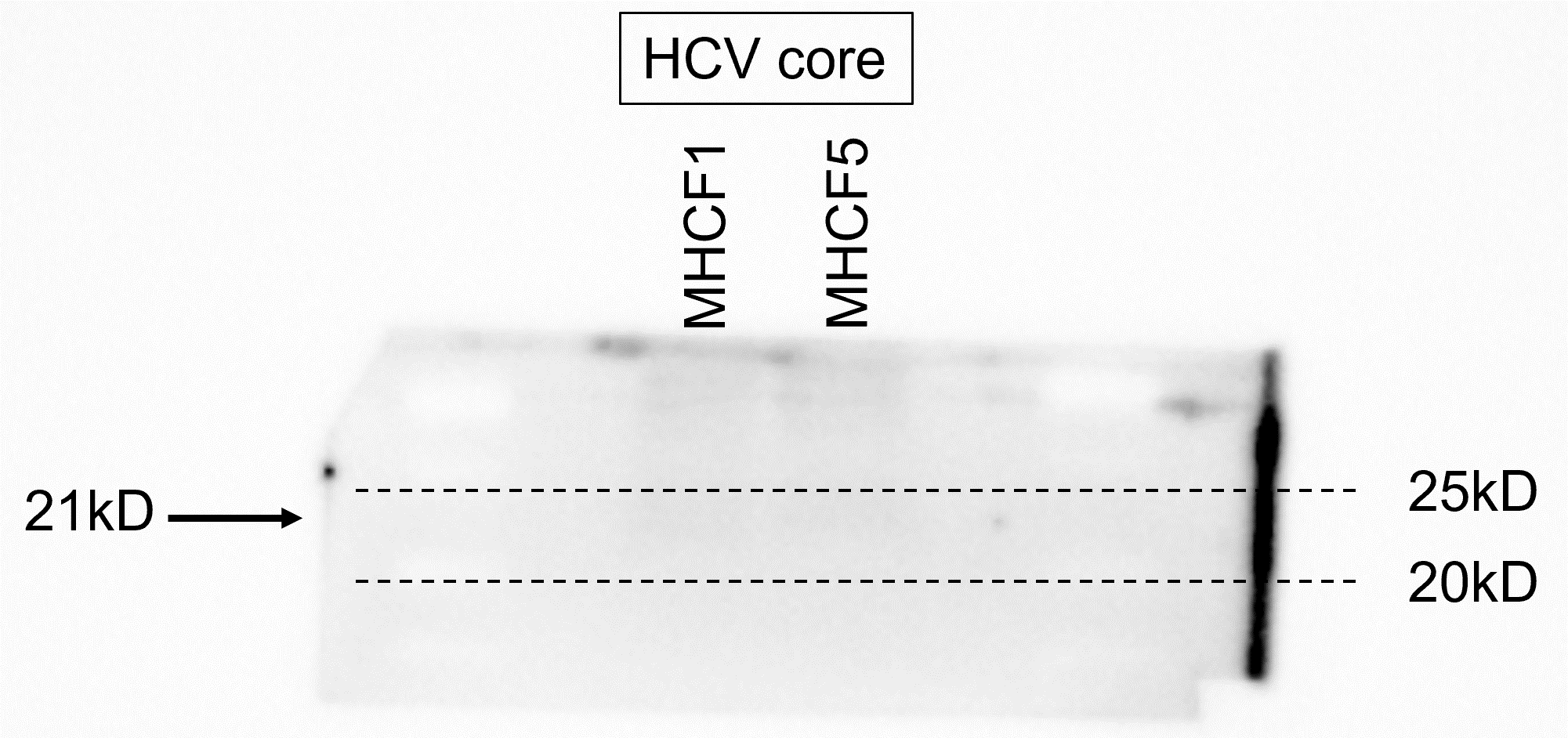


**A**


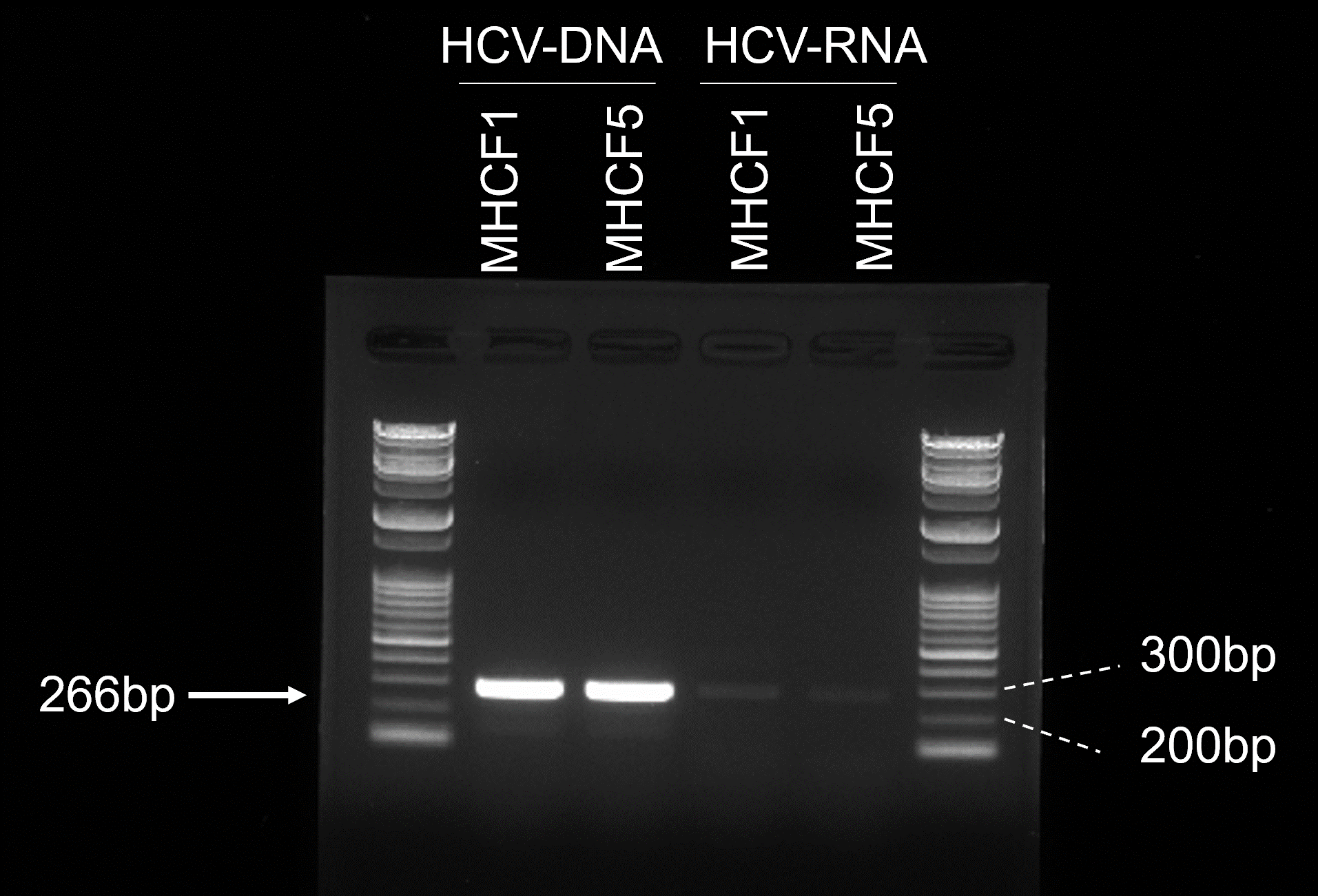


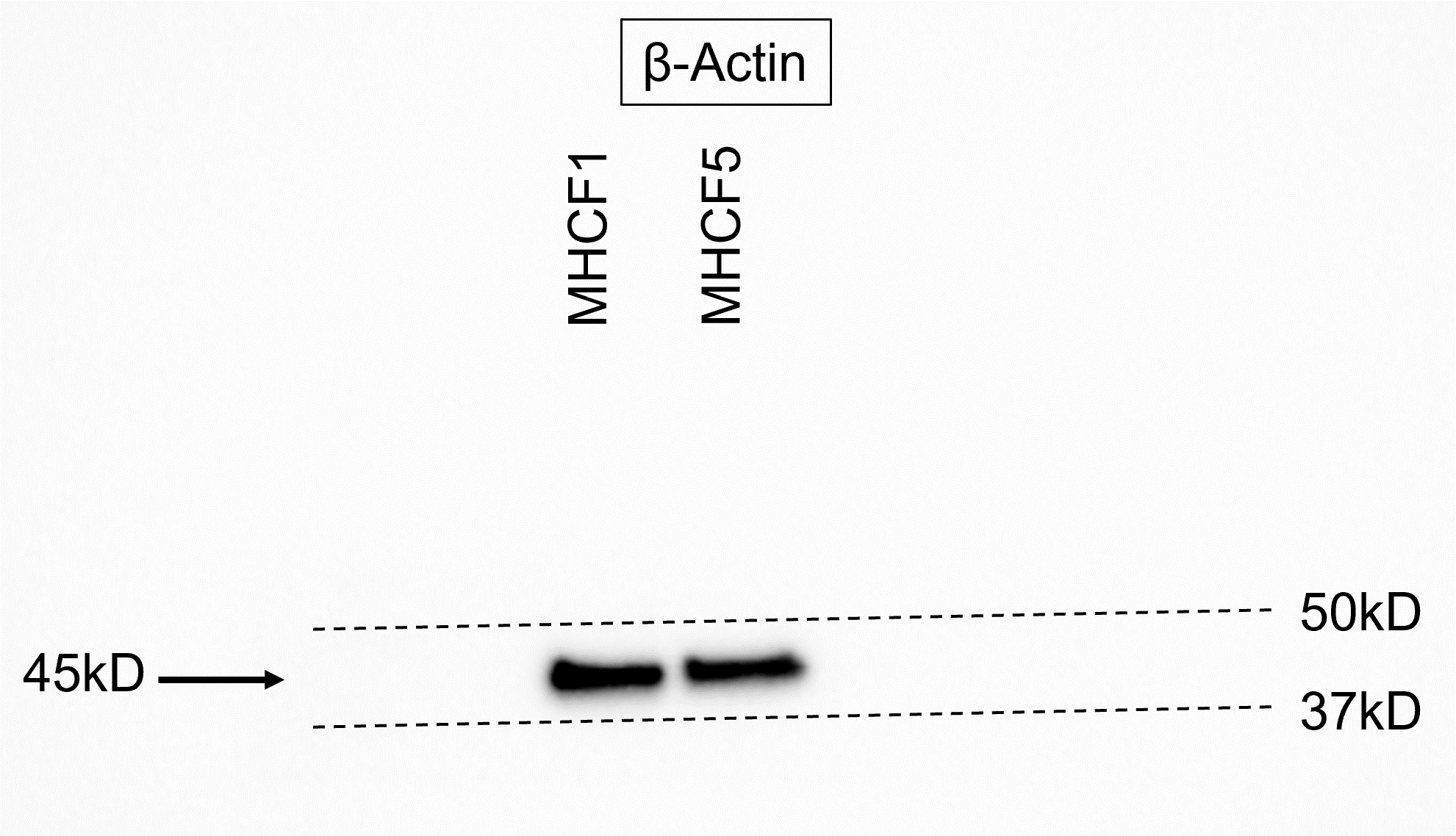


**Supplemental Figure 2.**

Expression of HCV-RNA and protein in MHCF1 and MHCF5 cells.

**A:** Total RNA was extracted from MHCF1 and MHCF5 cells. After DNase treatment, total RNA was subjected to reverse transcription and polymerase chain reaction (RT-PCR) using HCV core primers.

B: HCV core protein in MHCF1 and MHCF5 cells was assessed by western blotting.

**Supplemental Figure 3.**

Hepatocyte-specific gene expression in normal liver, MHCF1 cells, and MHCF5 cells.

**Supplemental Figure 4.**

Functional ontology enrichment analysis to compare the BioCarta Pathway process distribution of the differentially expressed genes.

**A**: Common upregulated or downregulated pathways in MHCF1 and MHCF5 cells compared with healthy liver. One-way hierarchical clustering of MHCF1 cells, MHCF5 cells, and healthy liver using differentially expressed pathway genes.

**B**: Differentially regulated pathways in MHCF1 and MHCF5 cells. One-way hierarchical clustering of MHCF1 and MHCF5 cells using differentially expressed pathway genes.
